# Development and Validation of the Arabic Short Assessment of Patient Satisfaction (Ar-SAPS) in General Practice Clinics of a Tertiary Academic Hospital

**DOI:** 10.3390/healthcare13192505

**Published:** 2025-10-02

**Authors:** Saad M. Alsaad, Abdulrahman A. Almuhaideb, Ahmed Alswailem, Max P. Jansen, Nasser M. AbuDujain, Khalid F. Alsadhan, Joud S. Almutairi, Abdullah A. Alrasheed, Turky H. Almigbal

**Affiliations:** 1 Department of Family and Community Medicine, College of Medicine, King Saud University, Riyadh P.O. Box 11495, Saudi Arabia; salsaad@ksu.edu.sa (S.M.A.); dr.khalidfm@gmail.com (K.F.A.); dr.jouds@hotmail.com (J.S.A.); aalrasheed1@ksu.edu.sa (A.A.A.); 2 College of Medicine, King Saud University, Riyadh P.O. Box 2925, Saudi Arabia; almuhaideb.abdulrahman@gmail.com (A.A.A.); ao.alswailem@gmail.com (A.A.); 3 Faculty of Social Sciences, Goethe University Frankfurt, D-60323 Frankfurt am Main, Germany; jansen@soz.uni-frankfurt.de; 4 Institute for Social Research (IfS), Goethe University Frankfurt, D-60323 Frankfurt am Main, Germany

**Keywords:** patient satisfaction, SAPS, validity, reliability, Arabic

## Abstract

Background and aim: Patient satisfaction is a critical indicator of healthcare quality, shaping treatment adherence, continuity of care, and the allocation of resources. The Short Assessment of Patient Satisfaction (SAPS) is a brief, reliable tool that is widely used internationally, but no validated Arabic version currently exists. Therefore, this study aimed to translate, culturally adapt, and validate the SAPS into Arabic for use in primary care clinics. Methods: We conducted a cross-sectional validation study at general practice clinics of a tertiary academic hospital in Riyadh, Saudi Arabia (June–August 2025). Consecutive Arabic-speaking patients aged 18–80 were recruited post-visit and completed a self-administered electronic survey including the Arabic Short Assessment of Patient Satisfaction (Ar-SAPS), PSQ-18, and PDRQ-9, as well as demographic and visit variables. Psychometric testing included internal consistency, test–retest reliability, construct validity, and factor analysis. Results: A total of 273 participants enrolled in our study. The Ar-SAPS demonstrated good reliability (Cronbach’s α = 0.789; McDonald’s ω = 0.882) and moderate test–retest stability (ICC = 0.634, *p* < 0.0001). Factor analysis supported a primarily unidimensional structure, with the first factor explaining 60.2% of variance. Most inter-item correlations were moderate to strong, except for item 6. Convergent validity was supported by significant correlations with the Arabic PDRQ-9 (*r* = 0.623, *p* < 0.001, CI [0.532, 0.713]) and PSQ-18 (*r* = 0.662, *p* < 0.001, CI [0.531, 0.793]), confirming consistency with established measures of patient satisfaction. Furthermore, it demonstrated excellent discriminative ability, with areas under the curve of 0.965 for overall satisfaction and 0.955 for willingness to recommend. Conclusion: The Ar-SAPS is valid and reliable for use to assess patient satisfaction.

## 1. Introduction

Patient satisfaction is widely recognized as a key performance indicator of primary health care quality and a cornerstone of patient-centered care [1]. It reflects how well services meet patients’ needs and expectations, influences whether patients return for care and adhere to treatment plans, and is frequently used to assess healthcare quality and inform decisions about hospital resource allocation and funding, alongside well-established determinants such as Diagnosis-Related Group (DRG) weight [2,3].

Institutions that consistently monitor and respond to patient experience data report measurable gains in clinical safety, effectiveness, and operational efficiency [4]. As a result, many health systems have incorporated patient satisfaction as a core component of routine quality improvement efforts, using patient feedback to assess performance, guide benchmarking, and inform service enhancements. In primary care settings, where physicians frequently serve as the first point of contact and maintain ongoing oversight of patient care, satisfaction metrics are particularly relevant. These tools help identify specific areas that require attention, such as excessive waiting times, suboptimal communication, and limited patient involvement in care decisions [5].

Numerous instruments have been developed to measure patient satisfaction; however, many are lengthy, disease-specific, or culturally constrained, which limits their routine use. The Short Assessment of Patient Satisfaction (SAPS) was designed to overcome these limitations: it is a seven-item, generic tool that can be completed in approximately one minute and demonstrates strong psychometric validity [6]. Each item reflects a core domain identified by the Institute of Medicine and the World Health Organization as essential to patient-centered care, treatment outcome, clarity of explanations, thoroughness, involvement in decision making, respect, time spent, and overall satisfaction. Its brevity facilitates use at the point of care, while its generic wording enables cross-specialty and cross-country benchmarking. Recent studies in Canada, the United Kingdom, Australia, and Southeast Asia have confirmed both its sensitivity to differences in care quality and responsiveness to quality-improvement interventions [7,8].

Measuring patient satisfaction in the patient’s native language preserves the intended meaning of key constructs [9]. In Arab healthcare systems, communication quality and waiting time [10] consistently emerge as dominant drivers of satisfaction, with additional effects from patient-clinician language concordance [11]. Accordingly, rigorously validated Arabic instruments are essential for capturing these nuances and enabling fair benchmarking across clinics and countries [12].

Although the SAPS is widely used internationally [6,13,14,15], assessing patient satisfaction in a patient’s native language enhances accuracy by capturing cultural nuances that are often overlooked in translation [16]. While Arabic adaptations of other instruments exist, none have yet addressed the SAPS, despite its strong international psychometric support [12,17,18]. In the absence of such a tool, healthcare providers across Arab nations often rely on locally developed or non-standardized surveys, which lack scientific validation and limit the comparability of patient feedback. This gap constrains efforts to benchmark care quality and align with international standards [16,18,19,20,21]. Accordingly, this study aimed to translate, culturally adapt, and validate the SAPS for use in Arabic, thereby providing a reliable instrument for measuring patient satisfaction and supporting healthcare improvement across the region.

## 2. Methodology

### 2.1. Study Design and Setting

We conducted a quantitative, cross-sectional instrument-validation study in the family medicine and primary care clinics at King Saud University Medical City in Riyadh, Saudi Arabia, from June to August 2025. Consecutive patients attending follow-up appointments during routine clinic hours were approached immediately after their visit. Eligibility required native Arabic proficiency, age ≥18 years, and the capacity to provide informed consent and self-complete a brief survey. Patients unable to consent or complete the questionnaire, or those with communication barriers, were excluded.

### 2.2. Measures

#### 2.2.1. Short Assessment of Patient Satisfaction (SAPS)

The SAPS is a seven-item, generic patient-reported experience measure that covers clarity of explanations, thoroughness, involvement in decision-making, respect, time spent, outcome of treatment, and overall satisfaction. Developed by Hawthorne and colleagues in 2011, it was first published in 2014. Items use a 5-point Likert scale; negatively worded items are reverse-scored. The total score is the sum of items, with higher scores indicating greater satisfaction. Permission to reproduce, translate, and administer the SAPS was obtained from the copyright holder [6].

#### 2.2.2. Patient Satisfaction Questionnaire Short-Form (PSQ-18)

The PSQ-18, developed by Marshall et al. [22] as a brief version of the PSQ-III, is a generic patient-experience measure comprising 18 items that cover seven domains: general satisfaction, technical quality, interpersonal manner, communication, financial aspects, time with the doctor, and accessibility and convenience. Items are rated on a 5-point Likert scale; negatively worded items are reverse-scored so that higher values indicate greater satisfaction. In the original development paper, PSQ-18 showed acceptable internal consistency (Cronbach’s α: general satisfaction = 0.75; technical quality = 0.74; interpersonal manner = 0.66; communication = 0.64; financial aspects = 0.73; time with doctor = 0.77; accessibility/convenience = 0.75) [22]. We used the Arabic version published by Hegazy et al. (Egyptian Journal of Hospital Medicine, 2021), which demonstrated excellent internal consistency (Cronbach α = 0.98–0.99) and good construct validity in an adult Arabic-speaking sample [23].

#### 2.2.3. Patient–Doctor Relationship Questionnaire (PDRQ-9)

The PDRQ-9, developed by van der Feltz-Cornelis and team in 2004, provides a brief assessment of the therapeutic aspects of the patient–doctor relationship in primary care from the patient’s perspective [24]. The scale consists of 9 items; each rated on a 5-point Likert scale (1 = not at all appropriate to 5 = totally appropriate). Scores are typically analyzed as a single mean (or sum) score, with higher values indicating a better perceived relationship. The original paper considered a two-factor model but retained a single, unidimensional scale, demonstrating strong psychometric performance with Cronbach’s α = 0.94. Later studies confirmed this one-factor structure and its high reliability (Cronbach’s α = 0.94) [25,26]. We used the Arabic version by Hegazy et al. (2021), which showed excellent internal consistency (α = 0.97–0.98) and satisfactory validity in Arabic-speaking adults [23].

### 2.3. Translation and Cultural Adaptation

Permission to adapt SAPS was obtained from the copyright holder. The Arabic version (Ar-SAPS) was developed through a structured translation and cultural adaptation process [27]. Two bilingual forward translations were prepared: TL-1, adopting the Arabic phrasing published by Almehman et al. [28], and TL-2, produced independently by a professional translator with a strong medical background. The forward translations were reviewed by N.M.A., S.M.A., and A.A. Afterwards, an independent translator, blinded from the source, prepared a back translation to confirm that the Arabic wording was natural and conveyed the same intent as the original English version in routine clinical use. Face validity was assessed by presenting the Ar-SAPS to 15 fluent Arabic speakers, who provided minor suggestions regarding syntax and clarity, all of which were incorporated into the final Ar-SAPS available in the Supplementary Materials.

### 2.4. Data Collection and Variables

After the visit, the survey was administered electronically via the SurveyMonkey platform (www.surveymonkey.com) and took approximately 5–7 min to complete. To assess temporal stability, a subset of 39 participants repeated the Ar-SAPS after a short interval suitable for test–retest reliability. In addition to the Ar-SAPS, PSQ-18, PDRQ-9, and anchor questions, we collected demographic information (age, gender, marital status, and education level). No identifying information was stored; responses were coded and handled securely.

### 2.5. Ethical Consideration

Ethics approval was granted by the College of Medicine, King Saud University IRB (No E-25-9584, Ref. No. 25/0311/IRB). The study adhered to the Declaration of Helsinki and guidance from the Saudi National Committee of Bioethics. Participation was voluntary; written informed consent was obtained after the visit. Surveys were anonymous; data were de-identified at collection, stored on restricted servers, and reported only in aggregate. Permission to translate and adapt the SAPS into Arabic was secured beforehand; no incentives were offered.

### 2.6. Statistical Analysis

To evaluate the psychometric properties of the Ar-SAPS, we examined its reliability, internal consistency, and construct validity. Internal consistency was assessed using Cronbach’s alpha and McDonald’s omega. To ensure that translating the scale stepwise does not alter its underlying latent structure, a two-step factor analytic strategy combining exploratory factor analysis (EFA) and confirmatory factor analysis (CFA) was conducted. The suitability of the data for factor analysis was confirmed using the Kaiser–Meyer–Olkin (KMO) measure and Bartlett’s test of sphericity. Standardized item loadings were reviewed to ensure conceptual clarity and alignment with the latent construct. Convergent validity was examined through correlations with two established patient satisfaction instruments (PDRQ-9 and PSQ-18). Finally, test–retest reliability was assessed by re-administering the Ar-SAPS to a subset of participants two weeks after their initial survey. The scale was analyzed using Stata statistical software, version 1.6, and the additional omegacoef command.

## 3. Results

The study included 273 participants, comprising 35.9% males and 64.1% females. The mean age for males was 51.3 ± 14.2 years, compared to 45.3 ± 12.9 years for females. The marital status distribution showed that 71.1% of the individuals were married. In terms of education, nearly half of the participants (49.1%) held a university degree, while 23.4% had postgraduate qualifications, 17.2% had secondary education, and 10.3% had only primary education. Clinic visit patterns indicated that the majority were regular visitors (62.2% of males and 65.1% of females attended at least three times per year). Overall satisfaction levels were high, with 96.9% of males and 92.6% of females reporting being satisfied with their medical care. Likewise, most participants expressed a willingness to recommend the clinic, with 89.8% of males and 90.9% of females indicating they would do so (see Table 1).

To assess whether the Ar-SAPS operated equivalently across subgroups, we conducted a multigroup CFA by gender. Results demonstrated that both the configural and metric models fit the data well for both male and female respondents (see Appendix A Table A1). Importantly, constraining factor loadings to be equal across gender led to only trivial changes in model fit indices (ΔCFI = 0.001, ΔRMSEA = 0.002). These results support the conclusion that the SAPS functions equivalently for men and women.

The Ar-SAPS demonstrated acceptable to good internal consistency, with a Cronbach’s alpha of 0.79, corresponding to an average interitem covariance of 0.25. McDonald’s omega was 0.882, indicating good composite reliability. The intraclass correlation coefficient (ICC) was 0.63, reflecting moderate test–retest reliability, with the associated *p*-value < 0.0001.

**Figure 1:**
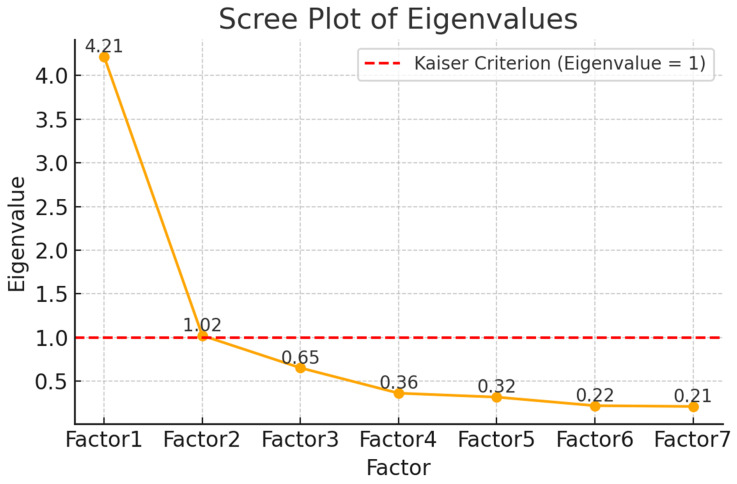
Scree plot of eigenvalues from the factor analysis of the Short Assessment of Patient Satisfaction (SAPS) items. The red dashed line indicates the Kaiser criterion (eigenvalue ≥ 1) for factor retention.

The principal component factor analysis without rotation revealed a primarily unidimensional model with eigenvalues greater than 1, accounting for a total of 60.20% of the variance in the Ar-SAPS scores and an eigenvalue of 4.21. The second component, which accounts for an additional 14.61% of the variance (eigenvalue of 1.02), suggests a potential secondary dimension—possibly linked to contextual or conditional satisfaction—though it remains secondary to the primary trait represented by the first component (see Figure 1). The sharp drop in eigenvalues after the second factor supports the retention of two factors based on the Kaiser criterion. The likelihood ratio (LR) test was highly significant, χ^2^(21) = 1132.82, *p* < 0.0001, indicating that the observed correlation matrix differs significantly from the identity matrix and that the data is suitable for factor analysis. Applying orthogonal varimax rotation improved interpretability while maintaining orthogonality between the factors. The rotated pattern matrix showed that Factor 1 was primarily defined by items saps1–saps5 and saps7, with high loadings ranging from 0.64 to 0.90, and moderate to low uniqueness values (0.18–0.51). Factor 2 was predominantly defined by item saps6 (loading = 0.98, uniqueness = 0.04), while also sharing smaller cross-loadings from saps5 (0.28). Factor 1 explained 59.94% of the variance, and Factor 2 explained 14.87%, consistent with the unrotated results. The factor rotation matrix confirmed minimal correlation between factors, supporting the appropriateness of orthogonal rotation. Results of a subsequent CFA support the finding of a primarily unidimensional factor structure (see Table A2 in the Appendix A). Additional CFA analyses excluding item 6 showed that model fit did not improve relative to the full seven-item version (RMSEA = 0.111; CFI = 0.973; TLI = 0.956; SRMR = 0.031). We therefore retained item 6 in order to preserve conceptual coverage and comparability with prior studies.

The inter-item correlation matrix for the Ar-SAPS shows that most item pairs demonstrate moderate to strong positive correlations (*r* ≈ 0.38–0.76), with nearly all reaching statistical significance (*p* < 0.05), indicating that the items measure related aspects of patient satisfaction. The strongest inter-item correlations are between SAPS2 and SAPS4 (*r* = 0.76, *p* < 0.001) and between SAPS3 and SAPS4 (*r* = 0.75, *p* < 0.001), suggesting substantial conceptual overlap. In contrast, saps6 exhibits weak inter-item correlations with most other items (*r* = 0.01–0.13), several of which are non-significant (e.g., saps6 with saps1: *r* = 0.04, *p* = 0.49) (Table 2).

**Figure 2:**
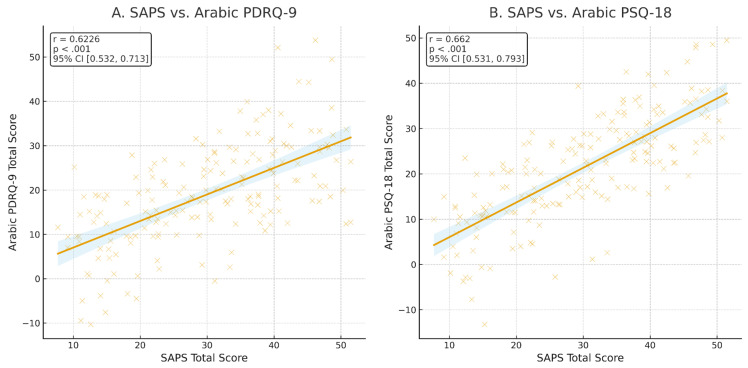
Scatter plots showing the correlation of Arabic SAPS total scores with (**A**) Arabic PDRQ-9 and (**B**) Arabic PSQ-18 total scores, indicating that higher patient satisfaction with healthcare services is moderately associated with a better patient–doctor relationship, suggesting a strong association between overall patient satisfaction and perceived quality of care.

**Figure 3:**
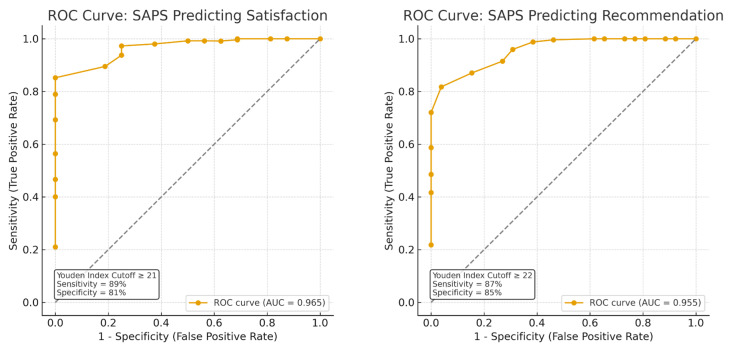
Receiver Operating Characteristic (ROC) curves showing the discriminative performance of the Short Assessment of Patient Satisfaction (SAPS) score for two outcomes: (**Left**) overall satisfaction and (**Right**) willingness to recommend the service. Each curve plots sensitivity (true positive rate) versus 1–specificity (false positive rate) across SAPS thresholds; the diagonal is the line of no discrimination (AUC = 0.5). Discrimination was outstanding for both outcomes, with AUCs of 0.97 for satisfaction and 0.96 for recommendation.

The construct validity of the Ar-SAPS was supported by its significant positive correlations with two established patient satisfaction instruments (see Figure 2). Specifically, scores on the Ar-SAPS demonstrated a moderate correlation with the Arabic PDRQ-9 (*r* = 0.62, *p* < 0.001, 95% CI [0.53, 0.71]), indicating that higher patient satisfaction scores were associated with better patient–doctor relationships. A stronger correlation was observed with the Arabic PSQ-18 (*r* = 0.66, *p* < 0.001, 95% CI [0.53, 0.79]), reflecting a close association between Ar-SAPS scores and perceived overall quality of care. These results indicate that the Ar-SAPS captures constructs consistent with established measures of patient satisfaction, with a particularly strong alignment to overall satisfaction and perceived care quality, thereby providing evidence for its convergent validity. Furthermore, the Ar-SAPS score demonstrated excellent discriminative ability for both overall satisfaction and willingness to recommend the service. The area under the ROC curve (AUC) was 0.97 for satisfaction and 0.96 for recommendation, indicating outstanding accuracy in distinguishing between satisfied versus unsatisfied participants and those who would or would not recommend the service. The ROC curves illustrated consistently high sensitivity and specificity across SAPS thresholds, with optimal cutoff points determined by the Youden Index cutoff of ≥21, (sensitivity = [89%], specificity = [81%]) and cutoff of ≥22, (sensitivity = [87%], specificity = [85%]) for overall satisfaction and recommending the clinic, respectively, providing a strong balance between true positive and false positive rates (see Figure 3).

## 4. Discussion

This study aimed to translate and validate an Arabic version of the SAPS questionnaire for use in outpatient clinics. The findings demonstrate that the Ar-SAPS is a valid and reliable instrument for assessing patient satisfaction with medical care. Its availability is particularly important in the Arabic language [12], spoken by over 450 million people in more than 25 countries, where standardized measures remain limited [29]. By providing a robust tool [6], the Ar-SAPS enables healthcare professionals and researchers to collect meaningful patient feedback, which may facilitate comparisons in similar tertiary primary-care settings; however, cross-country benchmarking requires further validation.

The Ar-SAPS demonstrated good reliability and structural validity and was generally consistent with the original and other adaptations. Internal consistency was strong (Cronbach’s α = 0.79; McDonald’s ω = 0.88), similar to the Australian original (α = 0.86) and Turkish adaptation (α = 0.87) [6,14]. The test–retest reliability was moderate (ICC = 0.63) [30], which was expected for a brief patient-reported measure, as some natural variation in responses would be likely after recent care. Factor analysis revealed additional evidence of the repeater instrument’s validity: the first component had an eigenvalue of 4.21 and accounted for 60.2% of the common variance, which far exceeds the typical 40% threshold for unidimensionality [31]. This ratio was higher than that of the Turkish adaptation (57.3%) [14], indicating that the Arabic items are slightly more clustered around satisfaction as a conceptual base. The original scale, validated using Mokken scalability and Rasch modelling, supported a clear one-factor structure. These findings confirm the structural integrity of Ar-SAPS and indicate that it is comparable to, and in some respects surpasses, previous generations.

Overall satisfaction was >90% (96.9% in men and 92.6% in women). This level reflects the tertiary academic setting, where specialist expertise, quick access to investigations, and a strong institutional reputation shape positive satisfaction. It also fits our respondents’ profile; many were frequent attenders (≥3 visits/year), indicating continuity with the same clinicians and established rapport. A further consideration is that social desirability bias in hospital-based surveys improves satisfaction. Therefore, the >90% figure is context-bound, useful for benchmarking similar tertiary clinics, but not proof of uniformly excellent care.

Comparable findings have been reported in other Saudi studies, where patient satisfaction was generally high across different specialties and service domains. For instance, patients at a Family Medicine Employee Clinic in Riyadh valued physician interactions but raised concerns about waiting times and appointment availability, while in tertiary care facilities professionalism and cleanliness were praised but administrative delays were criticized. Similarly, studies on nursing care and ophthalmology clinics highlighted strong communication and compassion from staff but persistent dissatisfaction with waiting times. In ambulatory care pharmacy services, most patients were satisfied with pharmacists’ professionalism and counseling, yet systematic follow-up was lacking. Collectively, these studies underscore a consistent pattern: while interpersonal aspects of care are well-regarded, structural and organizational issues—particularly long waits, inefficient appointment systems, and gaps in continuity—remain major challenges [32,33,34,35,36].

Construct validity of the Ar-SAPS was also well-supported. Fair to good correlations with the Arabic PSQ-18 and PDRQ-9 indicated convergence with broader measures of patient satisfaction and the doctor–patient relationship [37]. ROC analyses confirmed this evidence, demonstrating excellent discrimination for both overall satisfaction and willingness to recommend. Unlike the original Australian testing, this study prioritized psychometric modelling and known-groups validity, revealing that patients with depressive symptoms or poor medication adherence reported lower satisfaction, whereas those in private hospitals reported higher satisfaction. Overall, the Arabic validation not only confirmed unidimensionality but also enhanced construct validity through direct comparison with standard patient-reported outcomes, underscoring its representativeness for Arabic-speaking healthcare systems.

Moderately strong to strong correlations (*r* = 0.37–0.76, *p* < 0.05) were observed in interitem analyses, particularly among items related to explanations, thoroughness, and involvement in care, further supporting reliability and coherence. The sole exception to this was Item 6, concerning adequacy of time spent, which is reverse-worded and exhibits weaker, non-significant associations with the other items. This pattern is not a limitation; rather, it highlights the unique influence of time on patients’ experiential needs [38]. Consistent with short satisfaction scales developed and validated among non-Arab populations, affective and interpersonal-subjective items clustered together, while time-related factors formed a separate dimension. Our data indicates that perceived consultation speed can differ from other attributes and may disproportionately influence overall evaluations in healthcare systems with high patient loads and scheduling constraints. Retaining Item 6 enhances the Ar-SAPS by capturing this critical service dimension, ensuring satisfaction is not confined solely to communication aspects.

Nevertheless, excluding it would compromise comparability with the original SAPS. In contrast to the Turkish adaptation [14], in which all items displayed uniformly strong item–total correlations, and the original Australian validation that stated no ill-fitting items were detected in Rasch modelling, the Arabic version has revealed a cultural sensitivity to the way in which patients perceive consultation time. However, this does not compromise the overall credibility of the scale. Still, the addition of Item 6 adds content breadth, and robust AUCs against external anchors highlight the promise of Ar-SAPS for screening, benchmarking, and routine monitoring in QI programs.

Tracking patient satisfaction in this context helps uncover specific areas where care may fall short from the patient’s perspective (e.g., long wait times before appointments, unclear explanations during consultations, or a lack of involvement in making treatment decisions) [3]. For instance, when patients feel rushed or not listened to, they may become less engaged in their care and less likely to follow medical advice. By identifying these gaps, healthcare teams can implement targeted improvements, such as adjusting scheduling systems to reduce delays, training staff in effective communication skills, or developing tools that support shared decision-making. These efforts help create a more responsive and respectful environment for patients [13,39]. In turn, this leads to better experiences, stronger patient-provider relationships, and improved outcomes across various health measures.

Beyond its psychometric performance, the practical implications of the Ar-SAPS are significant. In daily clinical use, the ease of administration makes it feasible to routinely assess patient feedback without disrupting workflow. Furthermore, it would enable healthcare institutions to monitor patients’ needs more effectively, allowing better resource allocation.

This study has some limitations. It was limited to general practice clinics in a single tertiary hospital, which may not reflect other specialties or community settings. Electronic surveys completed after the visit may have introduced recall bias or selective participation. While a 1–2-week retest interval is standard practice, it may not fully capture long-term stability. Future studies with longer intervals are warranted. Although the ROC analyses yielded very high AUC values (0.96–0.97), these values may partly reflect ceiling effects in our sample because baseline satisfaction rates were generally high. While this could inflate the apparent discriminative performance, the consistency of the results across the subsamples indicates that overfitting is unlikely. Nevertheless, this should be considered when interpreting the findings. In addition, no universally accepted gold standard for patient satisfaction exists in Arabic; while comparisons with the PDRQ-9 and PSQ-18 are informative, they are not definitive benchmarks. However, despite the limitations, this study holds several strengths. The sample size (*n* = 273) exceeded recommended guidelines of 10–20 observations per item, ensuring robust psychometric testing [40]. Recruiting from general practice clinics provided a diverse mix of cases and demographics, supporting external validity within this setting. The translation and cultural adaptation process was rigorous, and multiple complementary indices (Cronbach’s alpha, McDonald’s omega, ICC, inter-item correlations, EFA, and ROC) were evaluated. Convergent validity was also confirmed against two established instruments (PDRQ-9 and PSQ-18). Lastly, electronic administration enhanced feasibility and scalability. Future studies should validate the Ar-SAPS in other outpatient specialties and in community or rural clinics to improve generalizability. Multicenter research across different Arab countries would also allow cross-cultural comparisons. Longer test–retest intervals and evaluations of responsiveness to quality improvement initiatives are needed, as well as assessments of measurement invariance across demographic groups.

## 5. Conclusions

The Ar-SAPS is a valid and psychometrically robust instrument for assessing outpatient satisfaction in Arabic-speaking settings. It demonstrates strong split-half internal consistency, test–retest reliability, and convergent validity, supporting its use in routine clinical practice and research, enabling consistent benchmarking, and promoting targeted improvements in patient-centred care throughout the Arab region.

## Figures and Tables

**Table 1:** Distribution of satisfaction/recommendation of the clinic by sociodemographic (*n* = 273).

	Overall Satisfied (Yes)	Overall Satisfied (No)	Recommend Clinic (Yes)	Recommend Clinic (No)
Gender				
Male (*n* = 98)	95 (34.8%)	3 (1.1%)	88 (32.23%)	10 (3.66%)
Female (*n* = 175)	162 (59.34%)	13 (4.76%)	159 (58.24%)	16 (5.86%)
Marital status				
Married (*n* = 194)	182 (66.67%)	12 (4.39%)	177 (64.84%)	17 (6.23%)
Unmarried (*n* = 79)	75 (27.47%)	4 (1.47%)	70 (25.64%)	9 (3.29%)
Education level				
≤High school (*n* = 75)	72 (26.37%)	3 (1.1%)	68 (24.91%)	7 (2.56%)
≥Bachelor (*n* = 198)	185 (67.77%)	13 (4.76%)	179 (65.57%)	19 (6.96%)

**Table 2:** Inter-item Correlation Matrix of Ar-SAPS.

	Ar-SAPS 1	Ar-SAPS 2	Ar-SAPS 3	Ar-SAPS 4	Ar-SAPS 5	Ar-SAPS 6	Ar-SAPS 7
Ar-SAPS 1	1.0						
Ar-SAPS 2	0.72 *	1.0					
Ar-SAPS 3	0.65 *	0.73 *	1.0				
Ar-SAPS 4	0.74 *	0.76 *	0.75 *	1.0			
Ar-SAPS 5	0.38 *	0.56 *	0.50 *	0.52 *	1.0		
Ar-SAPS 6	0.04	0.06	0.01	0.03	0.13	1.0	
Ar-SAPS 7	0.71 *	0.65 *	0.68 *	0.71 *	0.48 *	0.03	1.0

* Reflect significant *p*-value (<0.05).

## Data Availability

The data used in this study is available upon reasonable request from the corresponding author.

## Supplementary Materials

The following supporting information can be downloaded at: https://www.mdpi.com/article/10.3390/healthcare13192505/s1. The final Ar-SAPS.

## Abbreviations

SAPS	Short Assessment of Patient Satisfaction
AUC	Area Under the Curve
CI	Confidence Interval
EFA	Exploratory Factor Analysis
ICC	Intraclass Correlation Coefficient
IRB	Institutional Review Board
KMO	Kaiser–Meyer–Olkin
LR	Likelihood Ratio
PDRQ-9	Patient–Doctor Relationship Questionnaire (9 items)
PSQ-18	Patient Satisfaction Questionnaire Short-Form (18 items)
QI	Quality Improvement
ROC	Receiver Operating Characteristic
CFA	Confirmatory Factor Analysis
RMSEA	Root Mean Square Error of Approximation
CFI	Comparative Fit Index
TLI	Tucker–Lewis Index
SRMR	Standardized Root Mean Square Residual

## Appendix A

**Table A1:** Multigroup CFA testing measurement invariance across gender.

Model	χ^2^(df), *p*	CFI	TLI	RMSEA [90% CI]	SRMR	ΔCFI	ΔRMSEA
Configural	72.68 (40), 0.001	0.971	0.970	0.078 [0.048–0.106]	0.061	–	–
Metric	72.68 (39), 0.0009	0.970	0.968	0.080 [0.050–0.108]	0.061	0.001	0.002

Note. Male: *n* = 98, Female: *n* = 175. Configural model: Loadings and intercepts free; metric model: Loadings constrained across groups.

**Table A2:** CFA for one-factor structure.

Item	Factor Loading (F1)	Std. Err.	z	*p*	95% CI	Residual Variance
Ar-SAPS 1	1.000 (constr.)	–	–	–	–	0.202
Ar-SAPS 2	0.897	0.053	17.06	0.000	0.794–0.999	0.120
Ar-SAPS 3	0.842	0.052	16.23	0.000	0.740–0.943	0.128
Ar-SAPS 4	1.041	0.057	18.11	0.000	0.928–1.153	0.113
Ar-SAPS 5	0.512	0.051	9.99	0.000	0.412–0.613	0.216
Ar-SAPS 6	0.100	0.133	0.75	0.454	−0.161–0.361	1.878
Ar-SAPS 7	1.105	0.071	15.63	0.000	0.966–1.243	0.278
