# Supplementary material for: Development and Validation of the Arabic Short Assessment of Patient Satisfaction (Ar-SAPS) in General Practice Clinics of a Tertiary Academic Hospital
Source: Healthcare (Basel). 2025 Oct 2;13(19):2505. doi: 10.3390/healthcare13192505 (PMC12524747; doi:10.3390/healthcare13192505)
Supplement: Supplementary file 1 [file healthcare-13-02505-s001.zip › healthcare-3879433-supplementary.pdf]

The Short Assessment of Patient Satisfaction (SAPS)

Background

The Short Assessment of Patient Satisfaction (SAPS) is a short, reliable and valid seven item scale that can be used to assess patient satisfaction with their treatment. In 2006 (Hawthorne 2006, Hawthorne et al., 2006) a study was undertaken to examine a number of the leading patient satisfaction measures with urinary incontinence patients. The items from all these patient satisfaction scales were pooled and the SAPS was developed by selecting the items with best measurement properties and the most comprehensive coverage of the domains of patient satisfaction. The SAPS consists seven items assessing the core domains of patient satisfaction which include treatment satisfaction, explanation of treatment results, clinician care, participation in medical decision-making, respect by the clinician, time with the clinician, and satisfaction with hospital/clinic care. Responses scales are 5-point scales (see below).

The SAPS has been validated in clinical settings (Hawthorne et al., 2006; Sansoni et al., 2011) with support from the Australian Government Department of Health and Ageing. These studies have shown that the SAPS is a valid and reliable measure of patient satisfaction. Reliability is Cronbach's alpha $\alpha = 0.85$; it correlates highly with other measures of patient satisfaction, and correlates well with other indicators of treatment outcomes.

The SAPS is a generic measure of patient satisfaction. Although it was developed and validated in continence settings it can be used in any service settings with any treatment group. This means patient satisfaction scores in different treatment settings can be compared. With only 7 items the SAPS is short and simple to use and score. Most patients will only take a minute to complete it.

Why Use a Standardised Measure of Patient Satisfaction?

This means you are using the same yardstick to assess all patients. The use of such measures can provide effective feedback to clinicians concerning the patient's view of the effectiveness of their treatments, and can assist in identifying ways to improve practice and to address patient concerns. It is also useful information to demonstrate the effectiveness of your service. Continence clinics treating incontinence patients should find it easy to use as an outcome evaluation measure in routine practice.

Instructions: After reading each question, circle the answer that best describes you. The order of the answers varies between the questions, so take a moment to read each question carefully.

We know that sometimes answers may not describe you exactly, so please pick the answer that *most closely describes you*.

When you have finished, please check that you have answered all questions.

1. How satisfied are you with the effect of your {treatment/care}?

- ☐ *Very satisfied* *0*
- ☐ *Satisfied.* *1*
- ☐ *Neither satisfied nor dissatisfied.* *2*
- ☐ *Dissatisfied.* *3*
- ☐ *Very dissatisfied.* *4*

2. How satisfied are you with the explanations the {doctor/other health professional} has given you about the results of your {treatment/care}?

- ☐ *Very dissatisfied.* *0*
- ☐ *Dissatisfied.* *1*
- ☐ *Neither satisfied nor dissatisfied.* *2*
- ☐ *Satisfied.* *3*
- ☐ *Very satisfied* *4*

3. The {doctor/other health professional} was very careful to check everything when examining you.

- ☐ *Strongly agree* *0*
- ☐ *Agree* *1*
- ☐ *Not sure* *2*
- ☐ *Disagree* *3*
- ☐ *Strongly disagree* *4*

4. How satisfied were you with the choices you had in decisions affecting your health care?

- ☐ *Very dissatisfied* *0*
- ☐ *Dissatisfied.* *1*
- ☐ *Neither satisfied nor dissatisfied.* *2*
- ☐ *Satisfied.* *3*
- ☐ *Very satisfied.* *4*

5. How much of the time did you feel respected by the {doctor/other health professional}?

- ☐ *All of the time* 0
- ☐ *Most of the time* 1
- ☐ *About half the time* 2
- ☐ *Some of the time* 3
- ☐ *None of the time* 4

6. The time you had with the {doctor/other health professional} was too short.

- ☐ *Strongly agree* 0
- ☐ *Agree* 1
- ☐ *Not sure* 2
- ☐ *Disagree* 3
- ☐ *Strongly disagree* 4

7. Are you satisfied with the care you received in the {hospital/clinic}?

- ☐ *Very satisfied* 0
- ☐ *Satisfied.* 1
- ☐ *Neither satisfied nor dissatisfied.* 2
- ☐ *Dissatisfied.* 3
- ☐ *Very dissatisfied.* 4

Scoring

1. Reverse the scores for items #1, #3, #5, #7
2. Sum all scores. The score range is from 0 (extremely dissatisfied) to 28 (extremely satisfied)

Interpreting Scores

The literature on patient satisfaction shows that between 70-90% of patients are satisfied with their health care. This should be kept in mind when interpreting SAPS scores. In general, SAPS scores can be interpreted as follows:

- 0 to 10 = Very dissatisfied. To obtain a score in this range, a person must have indicated that they are dissatisfied or very dissatisfied on four or more items. Any patient obtaining scores in this range is indicating that their health care has failed them badly and that they are in need of urgent help.
- 11 to 18 = Dissatisfied. To obtain a score in this range, a person must have indicated that they are dissatisfied or very dissatisfied on at least two items (i.e. two aspects of their health care), or that they have refused to endorse being very satisfied on any item. Patients obtaining scores in this range are indicating health care failure in several areas of their health care and are in need of help in these areas.
- 19 to 26 = Satisfied. To obtain a score in this range, a person must have indicated that they are very satisfied or satisfied on over half SAPS items (4/7). These patients should be asked about those areas of health care they found unsatisfactory and efforts made to improve such areas.
- 27 to 28 = Very satisfied. To obtain a score in this range, a person must have indicated they are very satisfied or satisfied on all seven SAPS items. These patients are indicating that all aspects of their health care have met or exceeded their expectations.

In a recent study (Sansoni et al., 2011) the average score for all patients receiving incontinence treatment (N = 139) was 21.96 (SD 4.85); for females it was 21.75 and for males it was 23.09.

Further Information

Further Information can be found at www.bladderbowel.gov.au or from the Mental Health Evaluation Unit (MHEU), Department of Psychiatry, University of Melbourne at www.psychiatry.unimelb.edu.au/centres-units/cpro/index.html. These websites have down loadable copies of the Patient Administration Form, the Registration Form and the Validation Report. The SAPS is available free of charge but permission for use should be sought from the MHEU at the web address above. Additional information can also be obtained from Associate Professor Graeme Hawthorne at graemeeh@unimelb.edu.au.

Relevant Reports

Sansoni J, Hawthorne G, Marosszeky N, Moore K, Fleming G and Owen E. (2011), *Technical Manual and Instructions for the Revised Incontinence and Patient Satisfaction Tools.* Centre for Health Service Development, University of Wollongong

Sansoni J, Hawthorne G, Marosszeky N, Moore K, Fleming G, and Owen E (2011), *Validation and Clinical Translation of the Revised Continence and Patient Satisfaction Tools: Final Report.* Centre for Health Service Development, University of Wollongong.

Hawthorne G, Sansoni J, Hayes L M, Marosszeky N and Sansoni E (2006), *Measuring Patient Satisfaction with Incontinence Treatment (Final Report)*. Centre for Health Service Development, University of Wollongong and the Department of Psychiatry, University of Melbourne.

Study funded by the Australian Government Department of Health and Ageing as part of the National Continence Management Strategy

التقييم الموجز لرضا المريض

<u>وصف الأداة وطريقة حساب النقاط</u>

التعليمات: بعد قراءة كل سؤال، ضع دائرة حول الإجابة التي تصف حالتك على أفضل وجه. يختلف ترتيب الإجابات في كل سؤال، فاقرأ كل سؤال بعناية وعلى مهل. نعلم أن الإجابات في بعض الأحيان قد لا تصف حالتك بالضبط، لذا فضلًا اختر الإجابة الأقرب إلى وصف حالتك. بعد الانتهاء، فضلًا تأكد من أنك أجبت عن جميع الأسئلة.

**١- ما مدى رضاك عن أثر العلاج أو الرعاية التي حصلت عليها؟**	راضٍ جدًا	راضٍ	محايد	غير راضٍ	غير راضٍ وبشدّة
	( )	( )	( )	( )	( )
**٢- ما مدى رضاك عن الشرح الذي قدمه لك (الطبيب أو مقدم الرعاية) حول نتائج العلاج أو الرعاية التي حصلت عليها؟**	راضٍ جدًا	راضٍ	محايد	غير راضٍ	غير راضٍ وبشدّة
	( )	( )	( )	( )	( )
**٣- كان (الطبيب أو مقدم الرعاية) حريص جدًا على التأكد من كل شيء أثناء فحصك.**	أتفق بشدّة	أتفق	غير متأكد	أعارض	أعارض بشدّة
	( )	( )	( )	( )	( )
**٤- ما مدى رضاك عن الخيارات العلاجية المقدمة لك التي تتعلق في رعايتك الصحية؟**	راضٍ جدًا	راضٍ	محايد	غير راضٍ	غير راضٍ وبشدّة
	( )	( )	( )	( )	( )
**٥- ما مدة الوقت الذي شعرت فيه بأن (الطبيب أو مقدم الرعاية) عاملك باحترام واحترافية؟**	طوال الوقت	معظم الوقت	حوالي نصف الوقت	بعض الوقت	لم يحدث ذلك
	( )	( )	( )	( )	( )
**٦- الوقت الذي قضيته مع (الطبيب أو مقدم الرعاية) لم يكن كافيًا للتعامل مع شكواك.**	أتفق بشدّة	أتفق	غير متأكد	أعارض	أعارض بشدّة
	( )	( )	( )	( )	( )
**٧- هل أنت راضٍ عن الرعاية المقدمة لك في (المستشفى أو العيادة)؟**	راضٍ جدًا	راضٍ	محايد	غير راضٍ	غير راضٍ وبشدّة
	( )	( )	( )	( )	( )

حساب النقاط ومفتاح الإجابات:

يُحسب لكل بند نقاط كما هو مكتوب عند الإجابة المختارة: (٠=٠)، (١=١)، (٢=٢)، (٣=٣)، (٤=٤). تُجمع نقاط البنود.
تتراوح النتيجة الإجمالية من صفر إلى ٢٨ حيث إن النقاط الأعلى تدل على درجات أعلى من رضا المريض.

**١- ما مدى رضاك عن أثر العلاج أو الرعاية التي حصلت عليها؟**	راضٍ جدًا	راضٍ	محايد	غير راضٍ	غير راضٍ وبشدّة
	٤	٣	٢	١	٠
**٢- ما مدى رضاك عن الشرح الذي قدمه لك (الطبيب أو مقدم الرعاية) حول نتائج العلاج أو الرعاية التي حصلت عليها؟**	راضٍ جدًا	راضٍ	محايد	غير راضٍ	غير راضٍ وبشدّة
	٤	٣	٢	١	٠
**٣- كان (الطبيب أو مقدم الرعاية) حريص جدًا على التأكد من كل شيء أثناء فحصك.**	أتفق بشدّة	أتفق	غير متأكد	أعارض	أعارض بشدّة
	٤	٣	٢	١	٠
**٤- ما مدى رضاك عن الخيارات المقدمة لك التي تتعلق في الرعاية الصحية المقدمة لك؟**	راضٍ جدًا	راضٍ	محايد	غير راضٍ	غير راضٍ وبشدّة
	٤	٣	٢	١	٠
**٥- ما نسبة الوقت الذي شعرت فيه بأن (الطبيب أو مقدم الرعاية) عاملك باحترام واحترافية؟**	طوال الوقت	معظم الوقت	حوالي نصف الوقت	بعض الوقت	لم يحدث ذلك
	٤	٣	٢	١	٠
**٦- الوقت الذي قضيته مع (الطبيب أو مقدم الرعاية) لم يكن كافيًا للتعامل مع شكواك.**	أتفق بشدّة	أتفق	غير متأكد	أعارض	أعارض بشدّة
	٠	١	٢	٣	٤
**٧- هل أنت راضٍ عن الرعاية المقدمة لك في (المستشفى أو العيادة)؟**	راضٍ جدًا	راضٍ	محايد	غير راضٍ	غير راضٍ وبشدّة
	٤	٣	٢	١	٠
